# Meta-analysis reveals the correlation of Notch signaling with non-small cell lung cancer progression and prognosis

**DOI:** 10.1038/srep10338

**Published:** 2015-05-21

**Authors:** Xun Yuan, Hua Wu, Hanxiao Xu, Na Han, Qian Chu, Shiying Yu, Yuan Chen, Kongming Wu

**Affiliations:** 1 Department of Oncology, Tongji Hospital of Tongji Medical College, Huazhong University of Science and Technology, 1095 Jiefang Avenue, Wuhan 430030, P.R. China

## Abstract

Various studies have assessed the clinicopathological and prognostic value of Notch1 and Notch3 expression in Non-small cell lung cancer (NSCLC), but their results remain controversial. This meta-analysis was conducted to address the above issues by using a total of 19 studies involving 3663 patients. The correlations between Notch1 and Notch3 expression and clinicopathological features and NSCLC prognosis were analyzed. The meta-analysis indicated that higher expression of Notch1 was associated with greater possibility of lymph node metastasis and higher TNM stages. Moreover, patients with Notch1 overexpression and Notch3 overexpression showed significantly poor overall survival (Notch1: HR, 1.29; 95% CI, 1.06–1.57, p = 0.468 and I^2^ = 0.0%; Notch3: HR, 1.57; 95%CI, 1.04-2.36, p = 0.445 and I^2^ = 0.0%). Furthermore, there are statistically significant association between overall survival of NSCLC patients and the expression of Notch signaling ligand DLL3 and target gene HES1. Our meta-analysis supports that Notch signaling is a valuable bio-marker to predict progression and targeting Notch signaling could benefit subpopulation of NSCLC patients.

Non-small-cell lung cancer (NSCLC) is the most common cause of cancer-related deaths in the world and results in a serious problem to global health^1^. Despite advances in cancer biology as well as in diagnosis and treatment, progress in NSCLC therapy has been slow, leading to about 5% improvements in 5-year survival rates for the last 20 years^2^. Therefore, identifying the biomarkers is urgent to screen out high risk patients and predict outcome of NSCLC, in addition to the traditional clinicopathological features. Overwhelming evidence has proven that Notch signaling, comprised of four receptor isoforms (Notch1, Notch2, Notch3, Notch4) and five ligands members (Delta-like 1, Delta-like 3, Delta-like 4, Jagged-1 and Jagged-2), is crucial for cell proliferation, differentiation and apoptosis^3^. Earlier studies also discovered that deregulated Notch signaling led to various diseases, such as T cell leukemia and lung cancer^4^,^5^. In a transgenic mouse model, enhanced expression of Notch1 was found in the alveolar epithelium and increased abundance of Notch1 correlated with progressive from alveolar hyperplasia to pulmonary adenomas^6^. Subsequent study demonstrated that Notch signaling drove stemness and tumorigenicity of NSCLC^7^. The role of different Notch isoforms in NSCLC development is not clear, but Notch1 and Notch3 are believed to play a vital role^8^,^9^,^10^,^11^. Currently, one of the key points is to evaluate the value of Notch signaling as a molecular indicator of clinical features in NSCLC patients^12^,^13^,^14^,^15^.

A number of studies have examined the relationship between Notch1 and Notch3 expression and survival in patients with NSCLC. However, the prognostic value of Notch1 and Notch3 for NSCLC has yet to be confirmed. Some studies suggested that Notch1 and Notch3 overexpression was associated with poor prognosis in NSCLC^16^,^17^,^18^,^19^, but other researchers reported different results^20^. Besides, the ligands (Delta-like 1, Delta-like 3, Delta-like 4) and target genes, primarily involving in two families of helix-loop-helix transcription factors: HES (Hairy enhance of split) and HEY (Hairy/ enhancer of spit related with YRPW motif) targeting genes, were also used as biomarkers for the assessment of survival^21^,^22^,^23^. Given the uncertainty of causality and inconsistencies among studies, we performed a systematic review and a meta-analysis to evaluate the expression of Notch signaling members as predictive markers for clinicopathological parameters and outcomes of NSCLC.

## Results

### Search results

**Figure 1:**
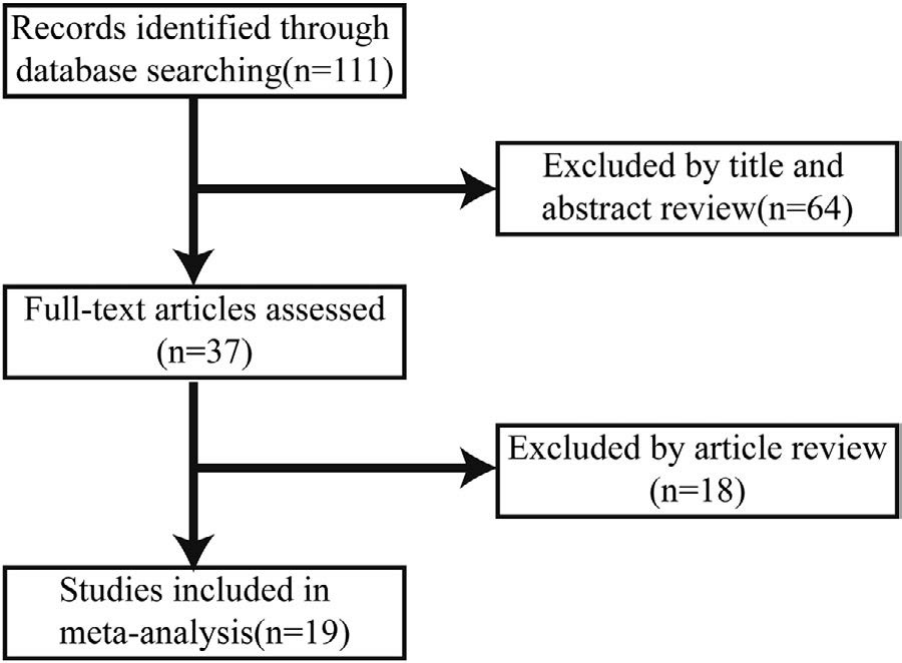
Flow diagram of article selection

The study flow diagram for the identification of eligible studies is shown in Fig. 1. 111 articles were found by our search strategy. After the article titles, abstracts and main texts were checked, 19 articles with 3663 cases were subjected to the criteria for this analysis. The features of the eligible studies are presented in Table 1. These studies mainly evaluated the correlation between the expression of Notch1 and Notch3 with clinical parameters for NSCLC, based on either clinicopathological features or prognostic factors. In the meantime, we conducted an analysis of the association between other members of Notch signaling with prognosis of NSCLC patients, including DLL1, DLL3, DLL4, HES1 and HEY1. Six of the 19 studies were from USA, 5 were from China, 2 were from Germany, 1 was from Canada, 1 was from Norway, 1 was from Netherland, 1 was from Japan, 1 was from Italy and 1 was from Korea. TNM stages I and II were classified as low stage, III and IV as high stage.

### Notch1 and Notch3 expression positively correlated with NSCLC progression

**Figure 2:**
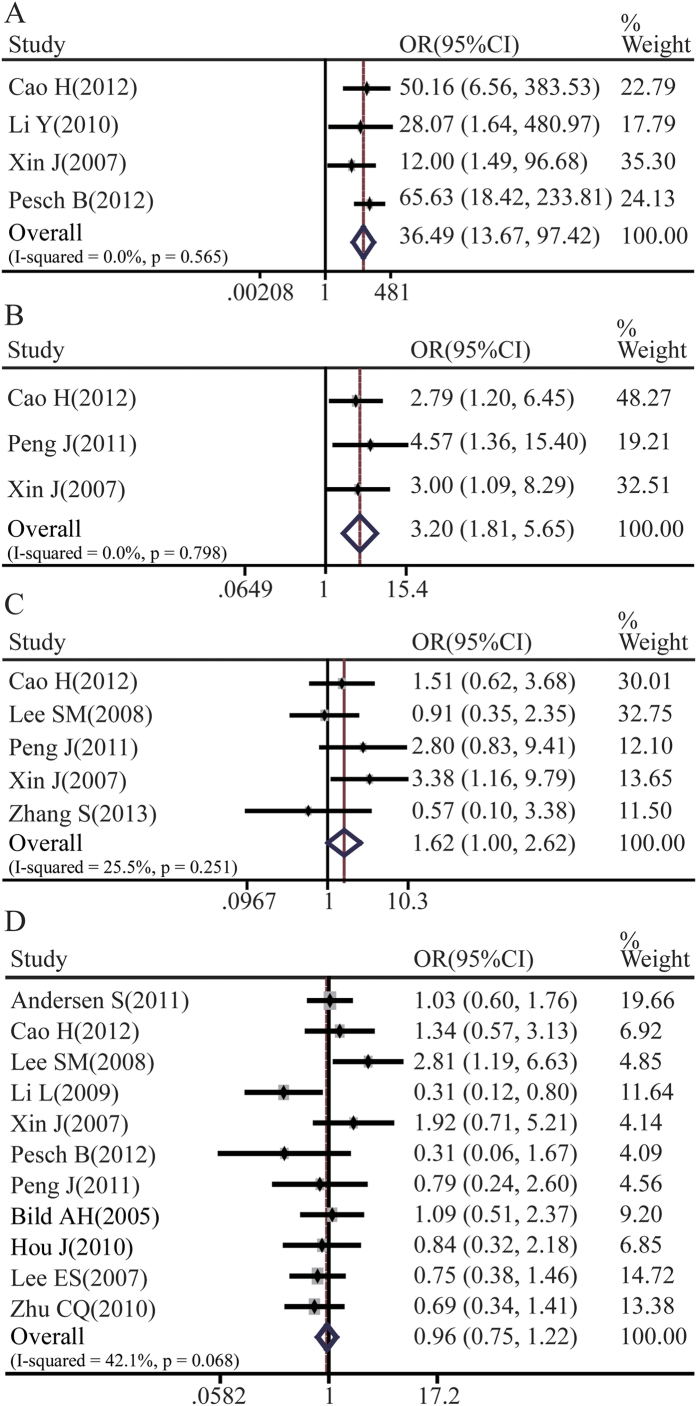
Forest plot of odds ratio (OR). CI, confidence interval. **A**. Relative Notch1 abundance of NSCLC in comparison to normal tissues. **B**. Association between Notch1 expression and NSCLC lymph node metastasis. **C**. Association between Notch1 abundance and NSCLC clinical stages. **D**. Relative expression of Notch1 in ADC compared to SCC.

There were a total of 13 references that assessed the correlation between Notch1 expression and tumor clinicopathological parameters. Our meta-analysis demonstrated that Notch1 expressions in NSCLC tissues were significantly higher than normal lung tissues (pooled OR = 36.49, 95%CI: 13.67-97.42, p = 0.565 and I^2^ = 0.0%) (Fig. 2A). Moreover, greater possibility of lymph node metastasis (LNM) and higher tumor stages were linked with high Notch1 expression in NSCLC (pooled OR = 3.20, 95%CI: 1.81-5.65, p = 0.798 and I^2^ = 0.0%; pooled OR = 1.62, 95%CI: 1.00-2.62, p = 0.251 and I^2^ = 25.5%) (Fig. 2B,C). However, there was no statistical association between Notch1 expression and two main histological types of NSCLC: adenocarcinoma (ADC) and squamous cell carcinoma (SCC) (pooled OR = 0.96, 95%CI: 0.75-1.22, p = 0.068 and I^2^ = 42.1%) (Fig. 2D). Although Notch 3 expression associated with lymph node metastasis, there was no correlation between Notch3 and tumor size. Similarly, there was no statistical difference between adenocarcinoma and squamous carcinoma (Supplementary figure).

### Impact of Notch1 expression on survival for NSCLC

**Figure 3:**
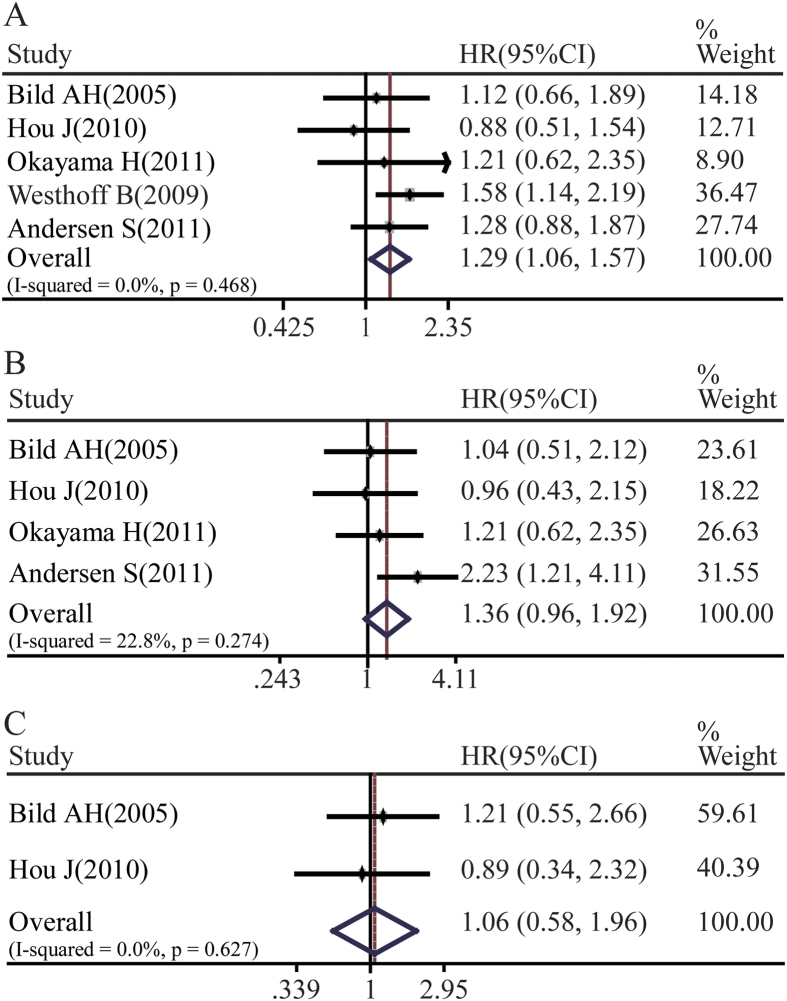
Forest plot of hazard ratio (HR). CI, confidence interval. **A**. Association between Notch1 and NSCLC OS. **B**. Association between Notch1 and ADC OS. **C**. Association between Notch1 and SCC OS.

The association between Notch1 expression and NSCLC prognosis was also analyzed. We found that Notch1 was correlated with the overall survival rate of NSCLC patients (pooled HR = 1.29, 95%CI: 1.06-1.57, p = 0.468 and I^2^ = 0.0%) (Fig. 3A). Although there was a tendency indicating Notch 1 expression associated with poor OS of adenocarcinoma, however there was no significant difference (pooled OR = 1.36, 95%CI: 0.96-1.92, p = 0.274 and I^2^ = 22.8%) (Fig. 3B). There was no significant relationship between Notch1 expression with lung squamous cell carcinoma overall survival rate (pooled OR = 1.06, 95%CI: 0.58-1.96, p = 0.627 and I^2^ = 0.0%) (Fig. 3C).

### Impact of Notch3 expression on survival for NSCLC

**Figure 4:**
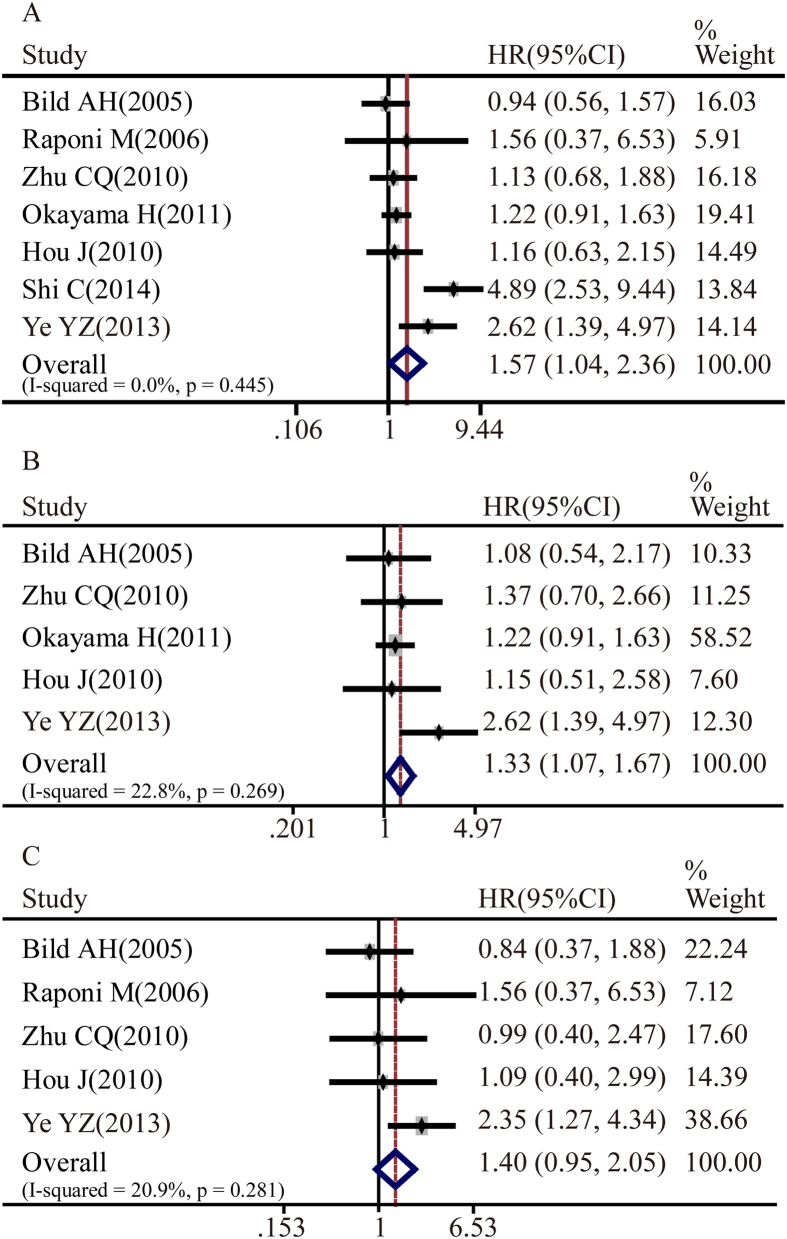
Forest plot of hazard ratio (HR). CI, confidence interval. **A**. Association between Notch3 and NSCLC OS. **B**. Association between Notch3 and ADC OS. **C**. Association between Notch3 and SCC OS.

There were a total of 9 studies that assessed the correlation between Notch3 expression and tumor clinicopathological parameters. Our meta-analysis demonstrated that Notch3 expressions in NSCLC tissues were significantly correlated with the overall survival rate of NSCLC patients (pooled HR, 1.57; 95%CI, 1.04-2.36, p = 0.445 and I^2^ = 0.0%) (Fig. 4A). In addition, Notch3 was also associated with the overall survival rate of lung adenocarcinoma patients (pooled HR = 1.33, 95%CI: 1.07-1.67, p = 0.269 and I^2^ = 22.8%) (Fig. 4B). There was no significant relationship between Notch3 expression with lung squamous cell carcinoma overall survival rate (pooled HR = 1.40, 95%CI: 0.95-2.05, p = 0.281 and I^2^ = 20.9%) (Fig. 4C).

### Impact of the expression of other members of Notch signaling on survival for NSCLC

**Figure 5:**
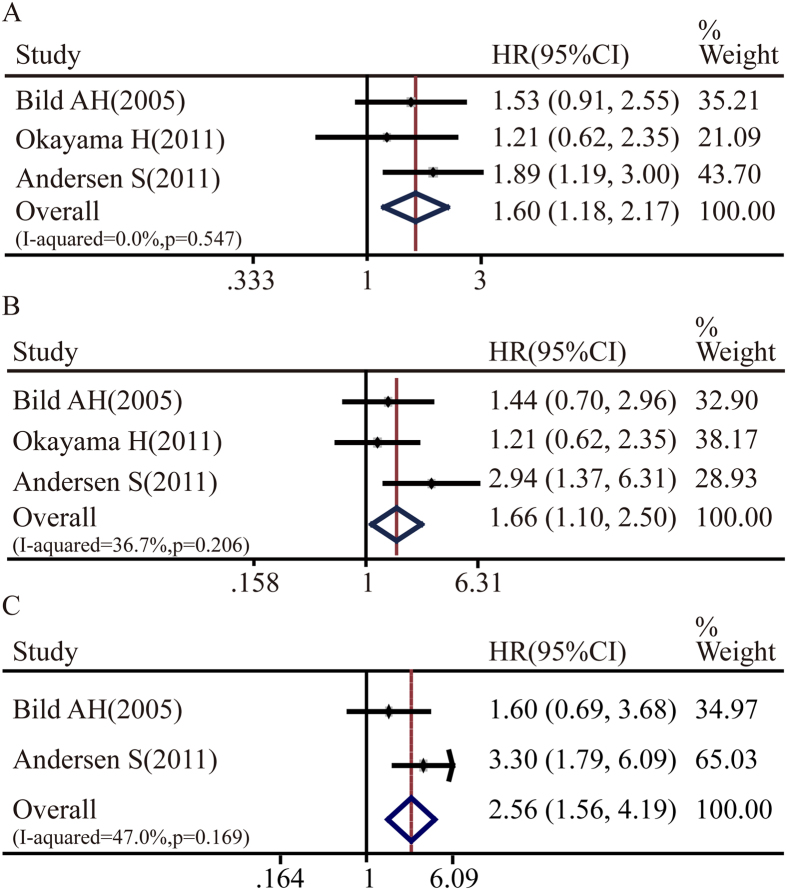
Forest plot of hazard ratio (HR). CI, confidence interval. **A**. Association between DLL4 and NSCLC OS. **B**. Association between DLL4 and ADC OS. **C**. Association between DLL4 and SCC OS.

Notch signaling is initiated by ligand binding to Notch receptor; therefore, we further analyzed expressions of Notch ligands and prognosis of NSCLC patients. As shown in Fig. 5, a statistically significant association between DLL4 expression and NSCLC overall survival rate was found (NSCLC: pooled HR = 1.60, 95%CI: 1.18-2.17, p = 0.547 and I^2^ = 0.0%; lung adenocarcinoma: pooled HR = 1.66, 95%CI: 1.10-2.50, p = 0.206 and I^2^ = 36.7%; lung squamous cell carcinoma: pooled HR = 2.56, 95%CI: 1.56-4.19, p = 0.169 and I^2^ = 47%). However, there was no significant relation between the expressions of DLL1 and DLL3 and overall survival of NSCLC patients.

**Figure 6:**
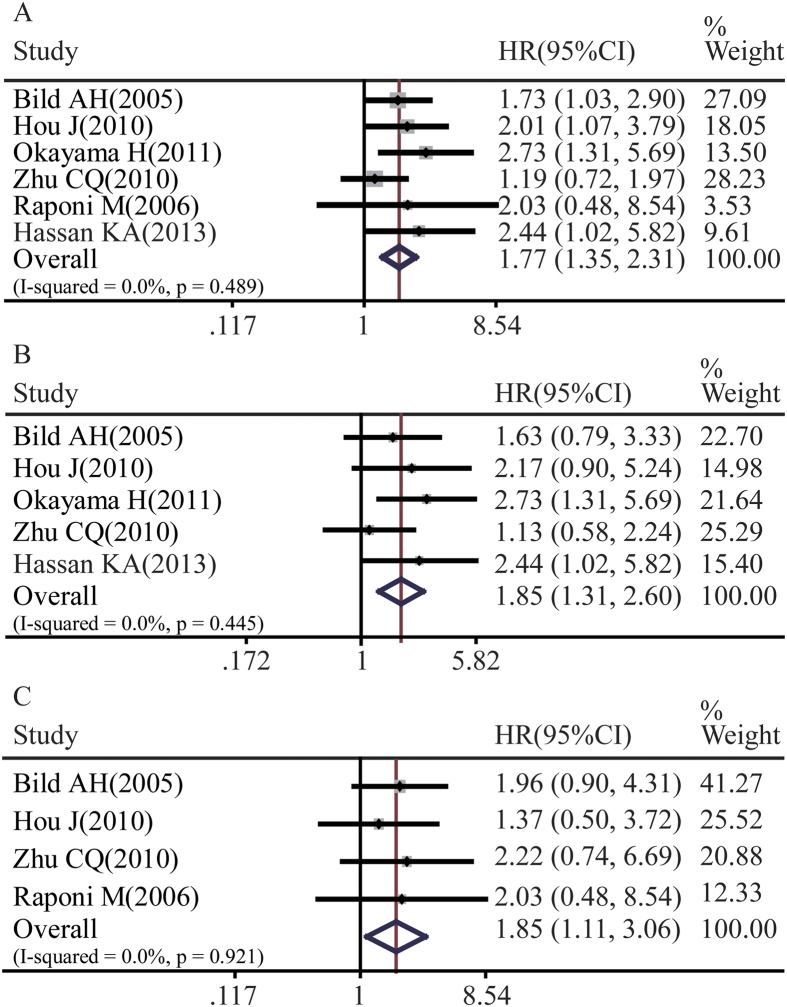
Forest plot of hazard ratio (HR). CI, confidence interval. **A**. Association between HES1 and NSCLC OS. **B**. Association between HES1 and ADC OS. **C**. Association between HES1 and SCC OS.

Next, we explored whether key targets of Notch1 and Notch3 could represent Notch signaling activity. The relationship between HES1 expression and NSCLC prognosis is illustrated in Fig. 6. Six studies including a total of 1087 patients demonstrated that HES1 overexpression was statistically associated with the overall survival rate of NSCLC patients (pooled HR = 1.77, 95%CI: 1.35-2.31, p = 0.489 and I^2^ = 0.0%) (Fig. 6A). In the subgroup analysis stratified by cancer histological types, HES1 expression was also associated with both lung adenocarcinoma overall survival rate (pooled HR = 1.85, 95%CI: 1.31-2.60, p = 0.445 and I^2^ = 0.0%) (Fig. 6B) and lung squamous cell carcinoma overall survival rate (pooled HR = 1.85, 95%CI: 1.11-3.06, p = 0.921 and I^2^ = 0.0%) (Fig. 6C).

**Figure 7:**
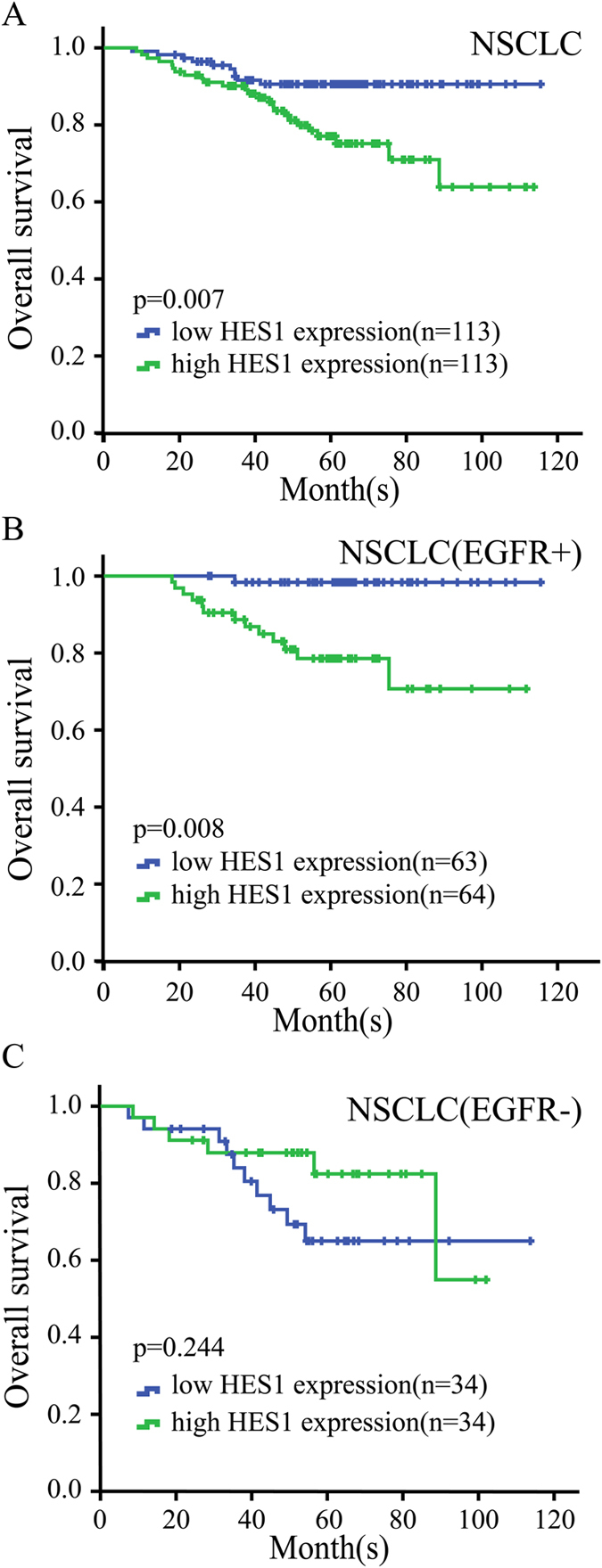
HES1 expression is correlated with overall survival of NSCLC patients(GSE31210). **A**. Overall survival rate was analyzed in 226 patients with NSCLC cancer, in relation to HES1 expression. **B**. Overall survival rate was analyzed in 127 patients with EGFR mutation+ NSCLC cancer, in relation to HES1 expression. **C**. Overall survival rate was analyzed in 68 patients with EGFR mutation- NSCLC cancer, in relation to HES1 expression.

Whole genomic expression profiles provide unbiased quantitative measure of mRNA abundance and have been proved to correlated with histo-pathological characteristics and predict prognosis^24^. To investigate whether HES1 mRNA abundance correlated with NSCLC survival, we analyzed published gene expression database of NSCLC with survival information. Kaplan –Meier survival curve of GSE31210 comprised of 226 NSCLC patients at stage I-II demonstrated that the overall survival rate was lower in patients with higher expression of HES1 (p = 0.007) (Fig. 7A). To explore whether mutation of EGFR interferes with HES1, NSCLC patients were subdivided into EGFR mutation and wild type (wt) group. The overall survival rate of patients with EGFR mutation was correlated with the abundance of HES1 expression (p = 0.008) (Fig. 7B). However, there is no statistical relation between HES1 expression and OS in EGFR wt patients (p = 0.244) (Fig. 7C). Together, analysis results from HES1 mRNA profile are in consisting with protein abundance, both indicating that higher Notch signaling activity predicts worse prognosis.

### Publication bias

Publication bias statistics were obtained using Begg’s test and Egger’s test, which did not indicate evidence of significant publication bias of Notch1 expression in NSCLC patients: NSCLC: Begg’s test, p = 0.296, Egger’s test, p = 0.591; LNM: Begg’s test, p = 1,Egger’s test, p = 0.553; TNM stages: Begg’s test, p = 1,Egger’s test, p = 0.713; lung adenocarcinoma: Begg’s test, p = 0.876, Egger’s test, p = 0.852; OS of NSCLC: Begg’s test, p = 0.221, Egger’s test, p = 0.123; OS of lung adenocarcinoma: Begg’s test, p = 0.089, Egger’s test, p = 0.148. Similar results were found for OS of Notch3, DLL4 and HES1 expression: Notch3 expression in NSCLC: Begg’s test, p = 0.072, Egger’s test, p = 0.97; Notch3 expression in adenocarcinoma: Begg’s test, p = 1,Egger’s test, p = 0.636; Notch3 expression in squamous cell carcinoma: Begg’s test, p = 0.806, Egger’s test, p = 0.402; DLL4 expression in NSCLC: Begg’s test, p = 0.296, Egger’s test, p = 0.176; DLL4 expression in adenocarcinoma: Begg’s test, p = 0.296,Egger’s test, p = 0.232; HES1 expression in NSCLC: Begg’s test, p = 0.806,Egger’s test, p = 0.291; HES1 expression in adenocarcinoma: Begg’s test, p = 0.462,Egger’s test, p = 0.259; HES1 expression in squamous cell carcinoma: Begg’s test, p = 1, Egger’s test, p = 0.9.

## Discussion

It is generally assumed that Notch1 and Notch3 activity were higher in advanced NSCLC and predicted poor prognosis^16^,^19^; However, opposite result was also reported in lung squamous carcinoma^10^. Precisely measuring the prognostic value of Notch1 and Notch3 may help to guide an individual therapy for NSCLC patients^24^. In our study, a combined analysis of 19 eligible clinical studies revealed a predictive value of Notch1 and Notch3 expression in NSCLC patients. The meta-analysis results suggested that Notch1 expression was significantly higher in lung cancer compared with normal tissues, and correlated with lymph node metastasis and TNM stages. However, Notch 3 was only associated with lymph node metastasis. In addition, our meta-analysis suggested that overexpression of Notch1 and Notch 3 could be a prognostic marker for OS.

Notch1 is commonly expressed in malignant cells from different types of cancer, participating in multiple functions, including motility, cell-cell connections and cell polarity^25^,^26^. Thus, Notch1 signaling could affect invasion, lymph node metastasis and TNM stages. Multiple studies have also demonstrated that Notch3 plays a role in the regulation of cellular proliferation, differentiation, apoptosis, as well as tumorigenesis^27^. Moreover, high Notch3 expression in lung cancer represented a higher possibility of being resistant to chemotherapy^28^. In support of clinical-pathological observation, experimental study demonstrated that high Notch activity enhanced tumor sphere formation and knockdown of Notch decreased cell proliferation and induced apoptotic cell death^29^. It has been shown that block Notch activity by GSI reduced xenograft growth of Notch abundant cells and decreased tumor incidence upon re-implantation. Correspondingly, molecular analysis confirmed the reduced expression of downstream effectors of Notch pathway from tumor xenografts of mice treated with GSI^23^, demonstrating a potent antitumor efficacy of Notch1 inhibitor. These findings suggest that GSI may provide novel therapies to improve the efficacy of conventional therapies by directly targeting the CSC, thus delaying tumor recurrence and improving cancer patient survival.

The identification of biomarkers for micro-metastases, disseminated tumors, and residual disease is critical for early detection and treatment of these diseases to prevent metastases and recurrence. To elucidate the correlation between the Notch signaling and NSCLC prognosis, analysis of Notch signaling cascade is necessary. Thus, the ligands and downstream target genes of Notch signaling were evaluated. In previous study, Dll4 was found to positively correlate with VEGFR1. Moreover, by up-regulation of Notch1 and VEGFR1, DLL4 played important roles in tumor angiogenesis and prognosis of lung cancer^30^. In addition, the activation of HES1 in NSCLC leaded to tumor cell growth and tumor progression^31^. Our meta-analysis is in agreement with previous report, showing that DLL4/HES1 were positively associated with poor OS of NSCLC patients.

Currently, new target therapies show a significantly improved progression-free survival (PFS) and relatively less toxicity in comparison with standard chemotherapy^2^. However, the inevitable resistance to target drugs limits theirs clinical efficiency. Identification of molecules cross-talk with target pathways will be critical for further improvements in NSCLC treatment. *In vivo* experimental studies observed significant antitumor activity in lung xenograft models accompanied with impaired tumor vasculature by reduced expression of several key angiogenic genes after the treatment of GSI RO4929097. Molecular analysis revealed Notch pathway target gene HES1 strongly corresponded with the therapeutic response. Thus, Notch pathway downstream genes could be used to predict the antitumor activity of RO4929097^32^.

EGFR mutations are one of the most important active genetic changes yet discovered in NSCLC. Both EGFR and Notch signaling are known to be deregulated in many human cancers. However, interactions of those two pathways are not well understood. It is observed that in human lung cancer cell line NCI-H292 cells, inhibition of the Notch pathway decreased both the Notch and EGFR/ERK pathways, associated with the reduction of proliferative cells. These results suggest that Notch signaling pathway has crosstalk with EGFR/ERK in human bronchial epithelial cells^33^. It can be proposed that GSIs might synergize with other signaling pathway inhibitors to treat NSCLC. For example, Notch 1 contributed to the acquired resistance to TKI and inhibition of Notch-1 resulted in effective response to EGFR target therapy (gefitinib)^34^.

Heterogeneity test is an essential part in meta-analysis. In this study, evidence of minor heterogeneities was observed; however, there was still some problems worth considering. Firstly, multicenter prospective studies based on large, homogeneous patient populations will be required. Another significant heterogeneity was likely due to the variations in assessing Notch signaling expression. The cutoff value was estimated in 5 studies by using the median Notch1 level measured by RT-PCR, remaining 12 articles measured by IHC. Some studies defined Notch1 expression based upon the percentage of immuno-positive cells or staining intensity, whereas other studies used a scoring system that combing those 2 factors together, causing the cutoff values for judging Notch1 expression diverse.

Lastly, publication bias is worth considering in meta-analysis. In this study, analyses of OS and other clinicopathological parameters did not show big variation. It should be noted that some limitations exist in the present meta-analysis. First, the number of included studies was relatively limited. Second, the applied method for detecting Notch signaling expression and the cutoff values were different in the included studies. Third, publication bias is still a concern. We tried to review all the relevant articles, but in some studies detailed description of raw data were not available.

All together, the meta-analysis provides evidence that Notch signaling is high activated in some NSCLC patients, which is associated with cancer progression and predicts bad prognosis. Preclinical models have proved the anti-tumor effects of GSIs. The therapeutic responses from early phase of clinical trials have observed in several types of cancer, including NSCLC. However, the dose -dependent adverse effects limited its clinical benefit 35. The identification and validation of of biomarkers associated with the hyperactivation of Notch signaling and correlated with treatment efficacy are important for a successful development of Notch pathway inhibitors. Incorporation of the specific biomarkers in future clinical trial may eventually demonstrate significant value of GSIs to subpopulation of NSCLC 36.

## Methods

### Literature search

We conducted a search of electronic databases PubMed, Embase and Cochrane library published up to October 1, 2014 using the search terms Notch (“Notch Receptors”, ”Notch Proteins”) and NSCLC (“Non-Small-Cell Lung Carcinoma,” “Non-Small Cell Lung Cancer”). The citation lists associated with the retrieved articles were also reviewed to identify additional relevant publications.

### Inclusion criteria

This meta-analysis collected data from randomized controlled studies (RCTs) or observational studies (case-control or cohort) that evaluated the relationship between Notch signaling expression and NSCLC parameters such as clinicopathological features and prognostic factors. Studies met the following criteria were included: a) patients recruited in the study were pathologically diagnosed to have NSCLC; b) Notch signaling expression was measured by immunohistochemistry (IHC) or real-time reverse transcription polymerase chain reaction (RT-PCR) in primary NSCLC tissue; c) the hazard ratio (HR) and corresponding 95% confidence interval (CI) were reported or could be statistically extracted from the study. When several articles were from the same patient population, the newest or most complete article was included.

### Data extraction

All data were abstracted by using a standardized data collection form, with information recorded as follows: first author’s last name, publication year, country of origin, histological type, tumor stage, number of cases and controls, detection methods, cutoff values for Notch1, Notch3, DLL4 and HES1. For articles without HRs, the statistical variables were calculated from published survival curves using methods described by Tierney *et al*^37^. We also reviewed Oncomine and found 5 independent human NSCLC microarray datasets with Notch signaling expression and survival data. Overall survival (OS) was evaluated by Cox proportional HRs and 95% CIs using these numerical data.

For observational studies, the Newcastle-Ottawa Quality Assessment Scale (NOS) was employed for assessing the quality of these studies. This scale is based on the identification of 8 sources of potential study bias that estimate patient selection, study comparability, and outcomes. Literature search, study selection, and data extraction were performed independently by two reviewers and disagreements among reviewers were resolved by discussion.

### Statistical analysis

Statistical analysis was conducted by the guidelines proposed by Meta-Analysis of Observational Studies^38^. The prognosis of NSCLC patients with expressions of Notch1, Notch 3, Notch ligands, Notch downstream target gene HES1 and HEY1was calculated by HRs and 95% CIs, respectively. Clinicopathological parameters included histological type, lymph node metastasis (LNM), TNM stage. Heterogeneity of the odds ratio (OR) and HR was appraised by using the Cochran Q and I^2^ test. A random-effect model was applied when p < 0.1 or I^2^ > 50%. When heterogeneity was absent, a fixed-effect model was employed. Publication bias was assessed by Begg’s rank correlation method and Egger’s weighted regression method. All p values were two tailed, and all analyses were carried out using STATA software package (version 12.0) (Stata Corp LP, College Station, TX, USA).

## Author Contributions

All authors contributed significantly to this work. K.W. and Q. C. designed and wrote the article. X.Y., H.W., H.X. and N. H. collected the data and performed the research study; S.Y. and Y.C. summarized and discussed the data. All authors reviewed this manuscript and approved the final draft.

## Additional Information

**How to cite this article**: Yuan, X. *et al.* Meta-analysis reveals the correlation of Notch signaling with non-small cell lung cancer progression and prognosis. *Sci. Rep.* **5**, 10338; doi: 10.1038/srep10338 (2015).

## Supplementary Material

Supplementary Information

## Figures and Tables

**Table 1:** Characteristics of studies included in the meta-analysis.

**First author**	**year**	**Country or area**	**Duration Month(s)**	**Histology**	**Stage**	**Patients n**	**Quality score**	**Detection**	**Cutoff values**
Andersen^16^	2011	Norway	216	NSCLC	I-IIIA	335	9	IHC (Notch1, DLL4)	Staining of H-score 0–1 vs. 2–3
Baumgart^8^	2010	Germany	NR	NSCLC	NR	46	9	IHC(Notch1)	Staining of H-score 0–1 vs. 2–3
Bild^17^	2005	USA	90	NSCLC	NR	111	9	RT-PCR(Notch1, DLL4, HES1)	median expression
Cao^9^	2012	China	NR	NSCLC	I-IIIA	131	8	IHC(Notch1)	Staining of H-score0–1 vs. 2–3
Hassan^23^	2013	USA	60	ADC	I-III	441	9	IHC(HES1)	median H-score
Hou^20^	2010	Netherland	130	NSCLC	NR	156	9	RT-PCR (Notch1, HES1)	median expression
Lee^24^	2008	Korea	NR	NSCLC	I-III	158	8	IHC(Notch1)	>10%
Li^10^	2009	USA	NR	NSCLC	NR	127	9	IHC(Notch1)	median H-score
Li^11^	2010	USA	NR	NSCLC	I-IV	395	8	IHC(Notch1)	Staining of H-score 0–1 vs. 2–3
Okayama^18^	2011	Japan	120	ADC	NR	226	9	RT-PCR(Notch1, DLL4, HES1)	median expression
Peng^12^	2011	China	NR	NSCLC	NR	55	7	IHC(Notch1)	median H-score
Pesch^13^	2012	Germany	NR	NSCLC	NR	68	7	IHC(Notch1)	Staining of H-score 0–1 vs. 2–3
Raponi^22^	2006	USA	60	SCC	I-IIIA	20	7	RT-PCR(HES1)	median expression
Shi^28^	2014	China	24	NSCLC	III-IV	594	8	IHC(Notch3)	median H-score
Westhoff^19^	2009	Italy	60	NSCLC	I-IV	420	9	IHC(Notch1)	median H-score
Xin^14^	2007	China	NR	NSCLC	I-III	80	8	IHC(Notch1)	Staining of H-score 0–1 vs. 2–3
Ye^27^	2013	China	60	NSCLC	I-IV	131	8	IHC(Notch3)	median H-score
Zhang^15^	2013	USA	NR	NSCLC	I-IV	41	8	IHC(Notch1)	Staining of H-score 0–1 vs. 2–3
Zhu^21^	2010	Canada	120	NSCLC	I-II	133	9	RT-PCR(HES1)	median expression

Abbreviation: NR, not reporting; IHC, immunohistochemistry; RT-PCR, real-time reverse transcription polymerase chain reaction; ADC, lung adenocarcinoma; SCC, lung squamous cell carcinoma.
