# Supplementary material for: Meta-analysis reveals the correlation of Notch signaling with non-small cell lung cancer progression and prognosis
Source: Sci Rep. 2015 May 21;5:10338. doi: 10.1038/srep10338 (PMC4440529; doi:10.1038/srep10338)
Supplement: Supplementary Information [file srep10338-s1.pdf]

Meta-analysis reveals the correlation of Notch signaling with non-small cell lung cancer progression and prognosis

Xun Yuan, Hua Wu, Hanxiao Xu, Na Han, Qian Chu, Shiying Yu, Yuan Chen, Kongming Wu

Department of Oncology, Tongji Hospital of Tongji Medical College,
Huazhong University of Science and Technology, 1095 Jiefang Avenue, Wuhan 430030, P.R. China.

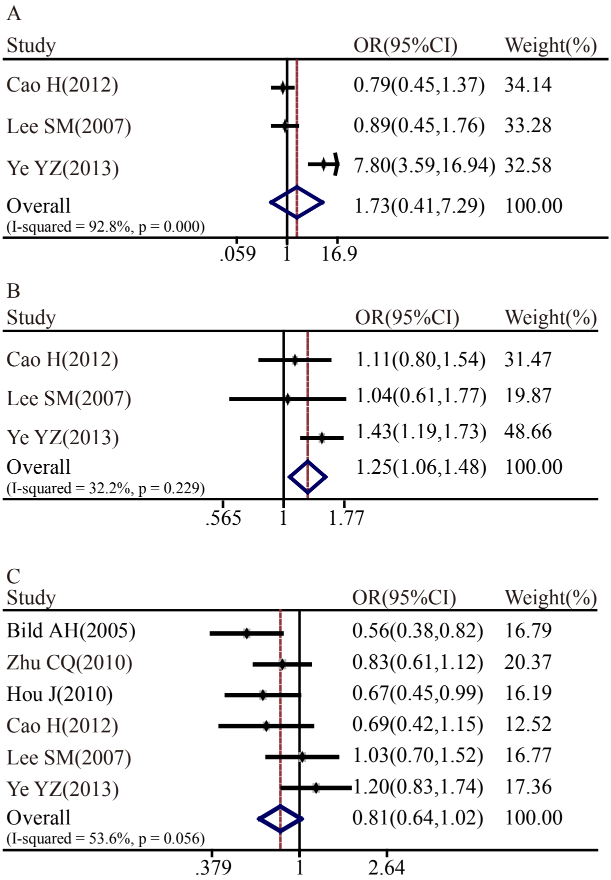

Figure S1. Forest plot of odds ratio (OR). CI, confidence interval.
A. Association between Notch3 expression and NSCLC clinical stages.
B. Association between Notch3 abundance and NSCLC lymph node metastasis.
C. Relative expression of Notch3 in ADC compared to SCC
